# CT Radiomics in Predicting EGFR Mutation in Non-small Cell Lung Cancer: A Single Institutional Study

**DOI:** 10.3389/fonc.2020.542957

**Published:** 2020-10-07

**Authors:** Shanshan Wu, Guiquan Shen, Jujiang Mao, Bo Gao

**Affiliations:** ^1^Department of Radiology, The Affiliated Hospital of Guizhou Medical University, Guiyang, China; ^2^Key Laboratory of Brain Imaging, Guizhou Medical University, Guiyang, China

**Keywords:** radiomics, non-small cell lung cancer, enhancement CT, EGFR, region of interest

## Abstract

**Objective:** To evaluate the value of CT radiomics in predicting the epidermal growth factor receptor (EGFR) mutation of patients with non-small cell lung cancer (NSCLC), and combing with the clinical characteristic to construct the prediction model.

**Methods:** Sixty-seven cases of NSCLC confirmed by pathology were enrolled. The pre-treatment chest CT enhanced images were used in Radiomics analysis. Two experienced radiologists delineated the region of interest (ROI) on open source software 3D-Slicer. The feature of ROI was extracted by Pyradiomics software package and a total of 849 features were extracted. By calculating Pearson correlation coefficient between pair-wise features and LASSO method for feature screening. The prediction model was constructed by logical regression, diagnostic efficacy of the model by the area under the receiver operating characteristic (ROC) curve was calculated.

**Results:** Based on clinical model and the radiomics model, the AUC under the ROC was 0.8387 and 0.8815, respectively. The model combining clinical and radiomics features perfect best, the AUC under the ROC was 0.9724, the sensitivity and specificity were 85.3 and 90.9%, respectively.

**Conclusions:** Compared with clinical features or radiomics features alone, the model constructed by combining clinical and pre-treatment chest enhanced CT features may show more utility for improved patient stratification in EGFR mutation and EGFR wild.

## Introduction

Lung cancer is accounting for 13% of the new cancer in the world (1), which is the main cause of cancer-related death. As a lung solid tumor, lung cancer shows a wide range of molecular heterogeneity. In the past decade, the treatment of non-small cell lung cancer (NSCLC) has evolved from previous cytotoxic chemotherapy to target therapy based on molecular changes, due to significant breakthrough in molecular research for its theranostics (2, 3). Small molecule tyrosine kinase inhibitors (TKIs) targeting specific EGFR mutations are the first targeted drugs for the treatment of NSCLC. Riely et al. (4) reported the response rate of EGFR-TKIs in patients with EGFR mutations (60–80%) was significantly higher than that in patients with EGFR wild-type or unknown mutation status (10–20%). In addition, a large number of clinical trials of Schuler et al. (5) found treatments with errotene, gefitinib, or afatinibthe in NSCLC with EGFR mutation would get a longer progress-free survival and higher objective response rate, compared with standard first-line chemotherapy. However, they also found if a non-EGFR mutant lung cancer patient was treated with targeted drugs such as gefitinib, the progression-free survival of the patient would be significantly shorter than those with first-line standard chemotherapy drugs (3). These data highlight the importance of accurately identifying the status of a patient's gene mutations in clinical to guide treatment.

For a clinical patient, the biopsy sample may be the only tumor material that can be used to detect the EGFR mutation status (6). However, tumor, it is possible to make the mutant DNA allele difficult to detect due to the sampling error and the observer difference, resulting in the occurrence of a false negative result (7). Second, living tissue examination is invasive, which increases the risk of complications. Last, genetic test is often expensive and some patients will not be able to afford it, which partly limits the clinical application of molecular test. Unfortunately, so far, there is still no stable, reliable clinical feature that can accurately predict the mutation status of EGFR. Radiogenomics has become a promising technique for identifying the gene phenotype in a tumor. As a new field of research, radiogenomics can integrate the image features and genome data of the disease, and to help explore the features of the image that can reflect the polymorphism or expression of the gene by digging the relation between the two. It is reported that using radiogenomics, Liu et al. (8) revealed EGFR mutation status of NSCLC was related to image features and could be predicted by five types of image feature. In this retrospective study, we tried to explore the relationships between CT imaging features and EGFR mutation in patients with NSCLC before treatment, and to construct a predictive model combined with clinical features.

## Materials and Methods

### Patient Population

All NSCLC cases confirmed by pathology from May 2017 to November 2018 were retrospectively collected in our hospital. The inclusion criteria were: (1) NSCLC confirmed by histopathology with complete clinical data; (2) Complete contrast enhancement chest CT imaging before treatment and follow-up images; (3) Complete genetic testing information. The exclusion criteria were: (1) Small cell lung cancer confirmed by histopathology; (2) Incomplete clinical data; (3) Image quality does not meet the requirements due to serious respiratory motion artifacts. Finally, 67 patients with EGFR detection information were analyzed, including 34 patients with positive EGFR mutation and 33 patients with negative EGFR mutation.

### Detection of EGFR Mutations

EGFR mutational analysis was performed on four tyrosine kinase domains (exons 18–21) that frequently mutated in lung cancer. The EGFR gene mutation was tested using human EGFR gene mutation detection kit (Beijing ACCB Biotechnology Company) using the Amplified refractory mutation system (ARMS) real-time technology.

### CT Scanning Protocols

Chest CT contrast enhancement examinations were performed using TOSHIBA CT (Aquilion PRIME TSX-302A, Japan), scanning parameters: tube voltage 120 KV, tube current 350 mA, field of view 390.0 × 390.0 mm, reconstruction thickness 5 mm, reconstruction interval 5 mm. Non-ionic iodine contrast agent (Bayer Pharmaceuticals, Berlin, Germany; Ompaque, Shanghai, GE) was intravenously injected at a rate of 2.5 ml/s using a high-pressure syringe at a dose of 1.3–1.5 ml/kg. The CT scanning was performed 50 s after the contrast injection.

### Radiomics Analysis

#### Image Segmentation and Feature Extraction

The segmentation of the region of interest (ROI) was performed by two experienced radiologist, all segmented in the open source software 3D-Slicer (9) (v4.10.0, download address: https://www.slicer.org/), the image data is saved in DICOM format. According to the three-dimensional (3D) volume of the tumor after segmentation, the tumor 3D features were extracted in the open source software package Pyradiomics (10) (https://github.com/Radiomics/pyradiomics/releases), and a total of 849 features were extracted for each patient. Among them, there are 14 shape features, 18 first-order statistical features (strength features), 74 texture features, and 743 wavelet features. Then, calculating the consistency of two expert extraction features, select the features with intra-class correlation coefficient (ICC) >0.75 for subsequent feature screening.

#### Feature Screening

**Figure 1:**
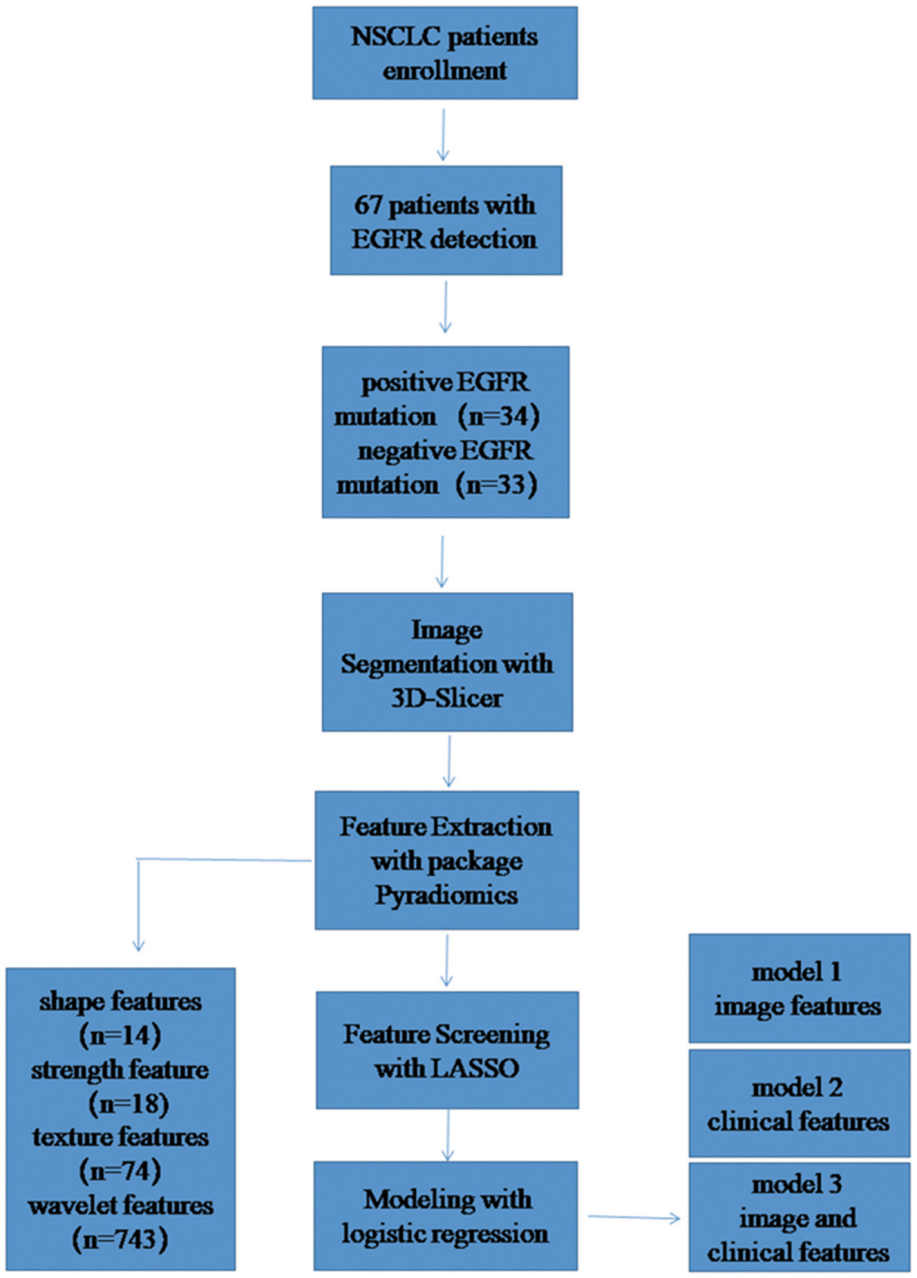
The study flow diagram.

After the feature consistency test, we were calculating the Pearson correlation coefficient between all the features, one is randomly excluded from each pair of features with a correlation coefficient >0.9, the other feature is selected, and so on, the redundancy of the feature is reduced (11).The feature is then screened by the least absolute shrinkage and selection operator (LASSO), LASSO is an accepted algorithm that has been used for feature selection in high-dimensional variables. Finally, the model is constructed by logistic regression method (12). LASSO method was applied to select the features that were most distinguishable and build a logistic regression model. A radiomic score (Rad_score) was obtained for each patient using features selected and weighted by the respective coefficients. The detailed study flow is shown in Figure 1.

#### Development of the Multivariable Prediction Model

A multivariable logistic regression model was built using clinical variable and radiomics features. All of these features were included in the development of a diagnostic model to predict EGFR mutation. We also developed a radiomic nomogram based on a multivariable logistic analysis. We used radiomic signature (Rad_signature) to represent the possibility for each patient, which was obtained by the LASSO regression model developed by radiomic features.

### Statistical Analysis

Statistical analysis was performed using R-3.4.4-win. In the clinical data of the patients, two independent sample *T-*tests were used to compare the age of the two samples, and the other clinical data were compared by chi-square test, *p* < 0.05 as the difference was statistically significant. The diagnostic efficacy of the radiomics model was analyzed by the receiver operating characteristic (ROC) curve of the subjects, and the area under curve (AUC) was calculated, and the sensitivity and specificity of the model were also calculated. The “glmnet” package is used to realize LASSO. In order to fit the excellent model better, the 10-fold cross-validation method is adopted (13). The nomogram was depicted based on the results of the multivariate analysis using the “rms” package in R. The “Hmisc” package was used to investigate the performance of the nomogram in concordance with the C-index.

## Results

### Patient

There were 34 patients with positive EGFR mutation and 33 patients with negative EGFR mutation, and there were significant differences in both cohorts detected in terms of smoking status, histological subtype, age, gender, or Rad_score. In the positive EGFR mutation, male patient comprised 32% (11/34) and female patients comprised 68% (23/34) of the total EGFR mutation cohort. The mean age was 53.1 ± 8.2 years. Adenocarcinoma was 94% (32/34) of all cases, squamous cell carcinoma was 6% (2/34). Smokers accounted for 24% (8/34) of patients, and non-smokers accounted for 76 (26/34). In the negative EGFR mutation, males were 82% (27/33) and females were 18% (6/33) of total patients. The mean age was 59.6 ± 7.8 years. Adenocarcinoma was 42% (14/33) of cases; squamous cell carcinoma was 58% (19/33). Smokers were 79% (26/33) of patients; non-smoker were 21 (7/33). More general information of patients was shown in Table 1.

**Table 1:** General information of 67 NSCLC patients.

**Subgroups**	**EGFR+**	**EGFR–**	**P-value**
Cases	34	33	
Age, mean ± SD	53.0882 ± 8.1997	59.6061 ± 7.8020	0.023
Gender			<0.001
Males	11 (32.35%)	27 (81.82%)	
Females	23 (67.65%)	6 (18.18%)	
Smoking			<0.001
Yes	8 (23.53%)	26 (78.79%)	
No	26 (76.47%)	7 (21.21%)	
Pathological stages			0.518
IIIb, IIIc	15 (44.12%)	12 (36.36%)	
Iva, IVb	19 (55.88%)	21 (63.64%)	
Histological types			<0.001
Adenocarcinoma	32 (94.12%)	14 (42.42%)	
Squamous cell carcinoma	2 (5.88%)	19 (57.58%)	
Rad_score (mean)	4.167	−3.947	<0.001

### Feature Consistency Testing and Screening

**Figure 2:**
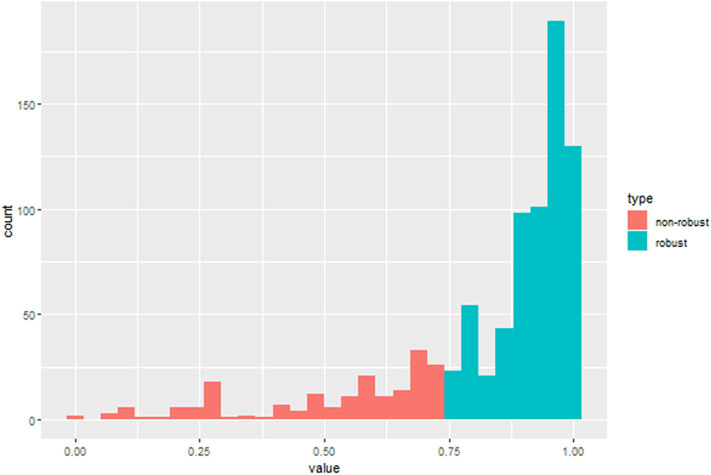
ICC histogram of radiomics features.

**Figure 3:**
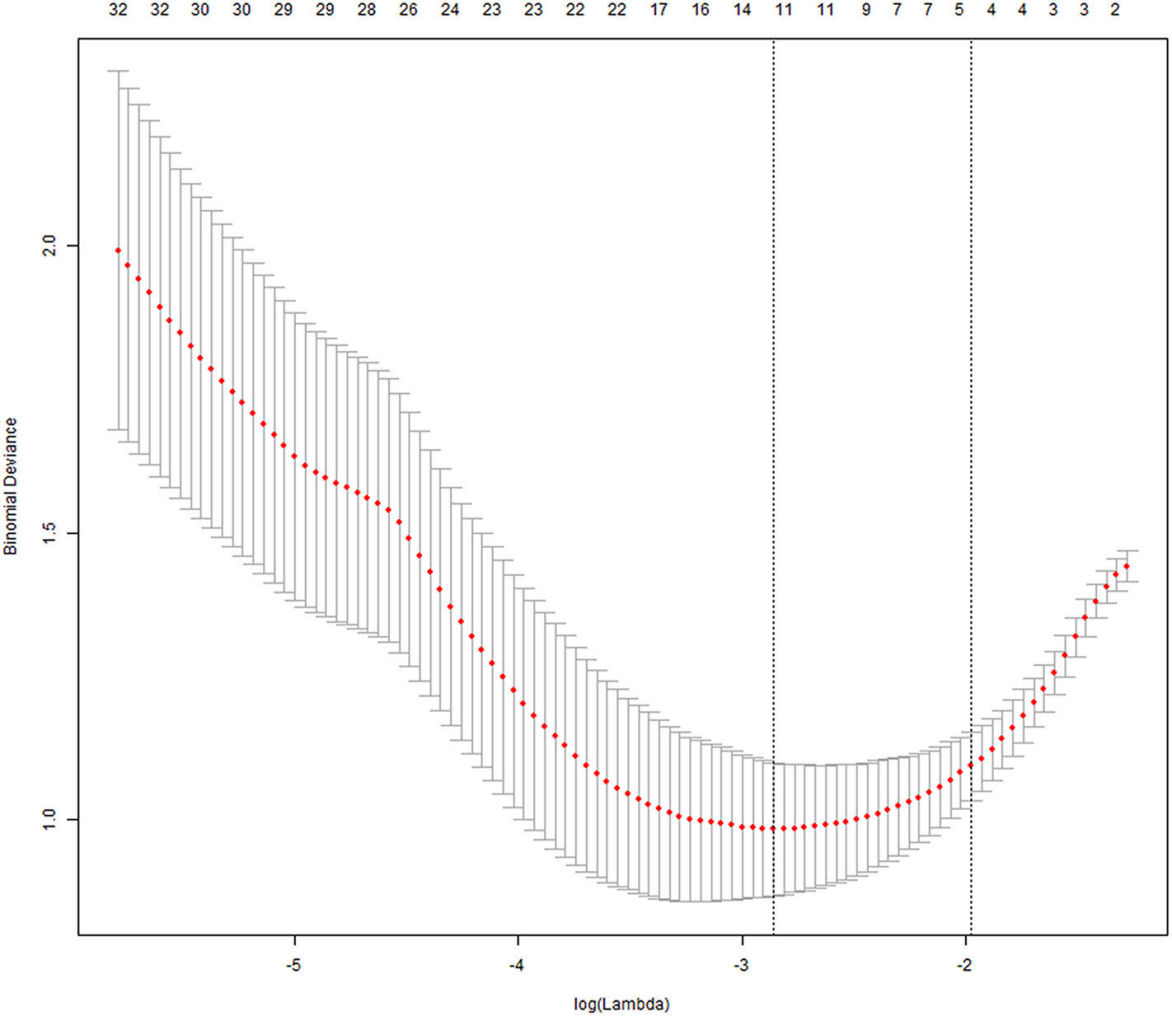
LASSO method for screening of radiomics features.

After the ICC analysis, there were 658 features ICC > 0.75 (Figure 2), these features combined with clinical variables will be used for subsequent feature screening. After Pearson correlation coefficient filtering, the number of features changed from 658 to 382. Based on the LASSO dimension reduction, as Figure 3, when the variable is equal to 12, the error classification value is lower, and 12 features related to the patient's EGFR mutation are selected to construct the LASSO logistic regression model.

### Development of the Multivariable Prediction Model

**Figure 4:**
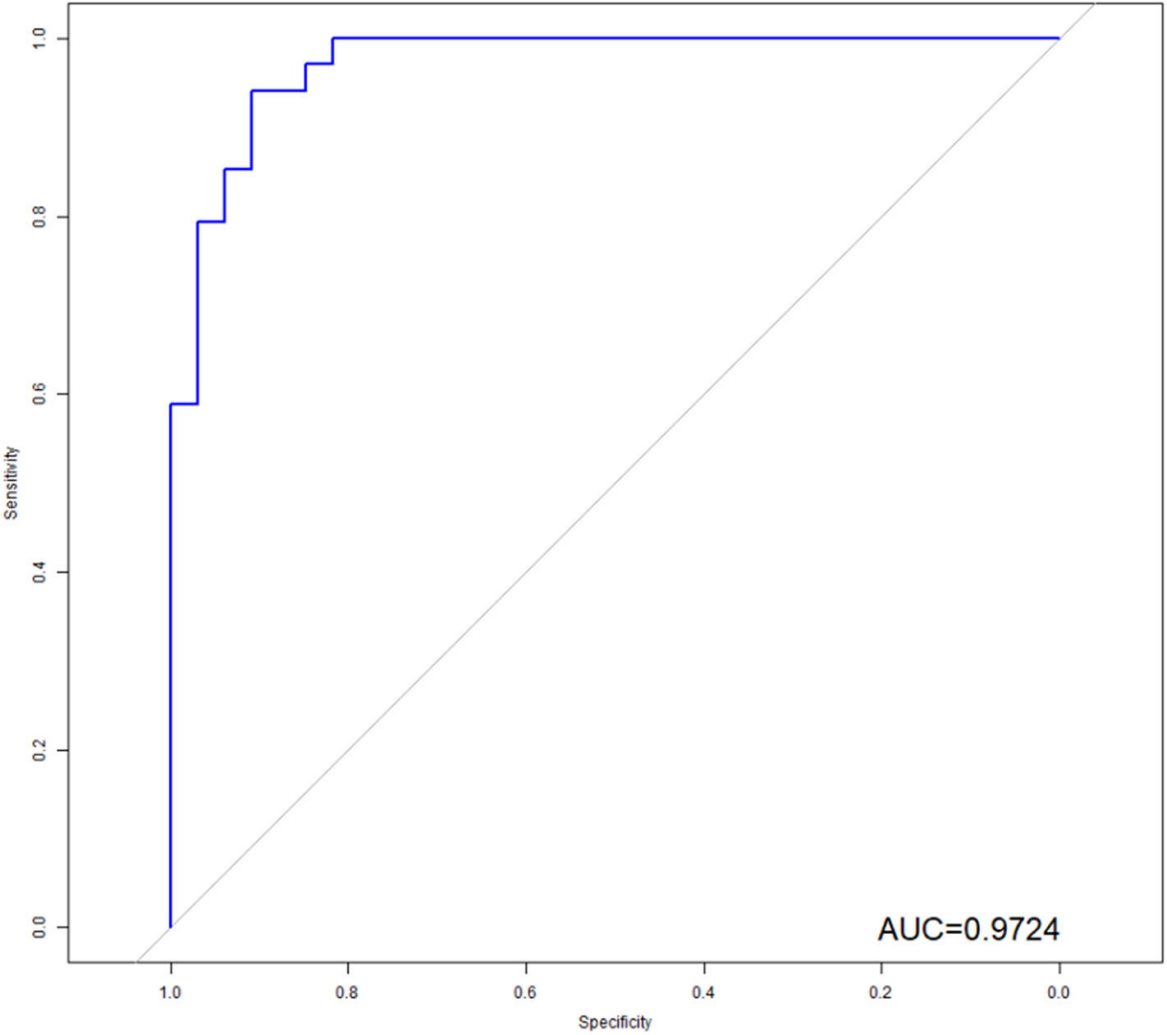
ROC analysis of the model. As shown above, radiomics features combined with clinical variables had the potential ability to predict the EGFR mutation, the AUC was 0.9724.

The LASSO logistic regression analysis (Figure 4) revealed that 10 radiomic features combined with two clinical features had the potential to build the prediction model for EGFR mutation by the training of 67 cases, which include Shape.Surfacevolume ratio; Gldm.dependencevariance; W_HLL_.glcm.idn; w_LHL_.ngtdm.Contrast; W_LHH_.gldm.Large_emhpasis; W_LHH_.gldm.Small_emphasis; W_HHL_.gldm.variance; W_HHL_.ngtdm.busyness; W_LLL_.gldm.variance; W_LLL_.glrlm.long_emphasis; smoking status and histological subtype. In addition, we calculated the sensitivity, specificity, positive predictive value, negative predictive value and accuracy to show the ability, the details are shown in Table 2.

**Table 2:** Diagnostic accuracy of prediction model.

**Sensibility**	**Specificity**	**Positive**	**Negative**	**Accuracy**
**%**	**%**	**predictive value**	**predictive value**	**%**
		**%**	**%**	
85.3	90.9	90.6	85.7	88.1
(29/34)	(30/33)	(29/32)	(30/35)	(59/67)

### ROC Curves Analysis for Radiomic Features and Clinical Predictors

**Figure 5:**
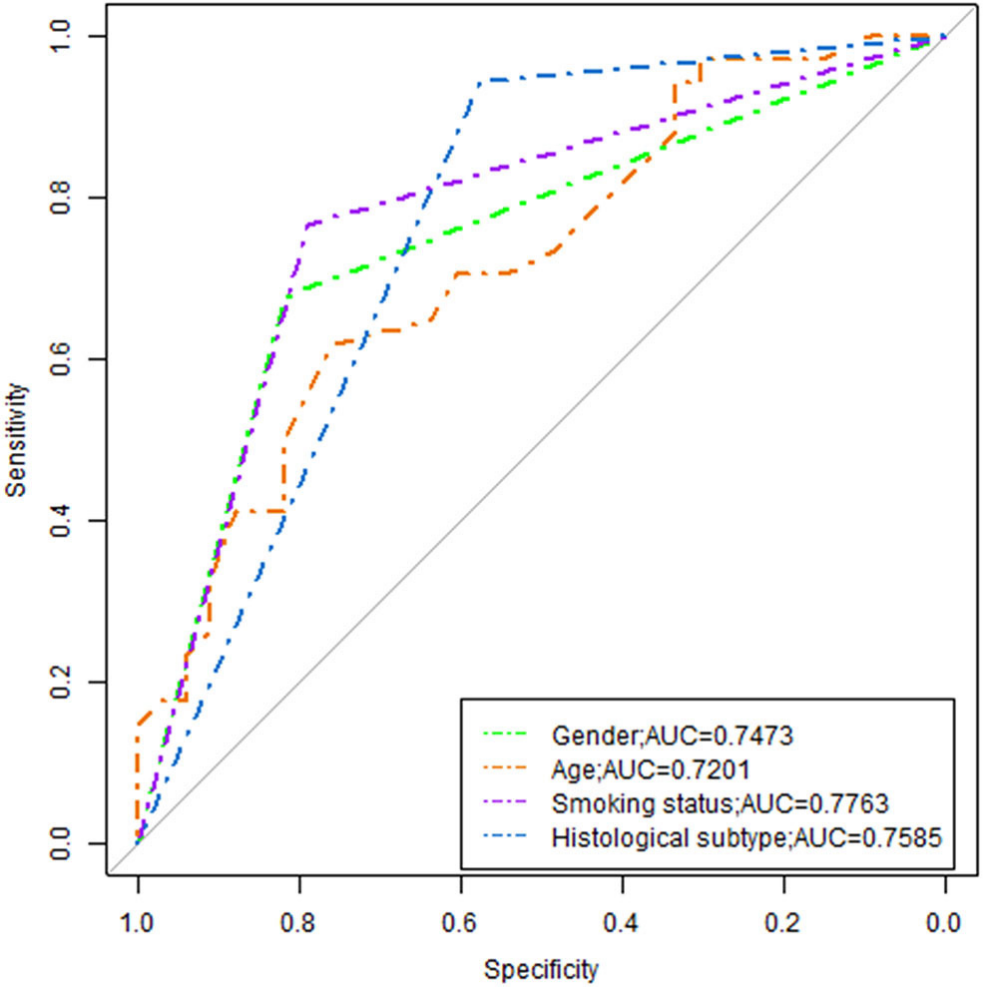
ROC analysis of the clinical variable.

The model revealed that gender, smoking status, clinical stage, and histological subtype were independent predictors of EGFR mutation. However, the model was merely developed by these features showing poor performance, the AUC ranged from 0.72 to 0.78 (Figure 5).

**Figure 6:**
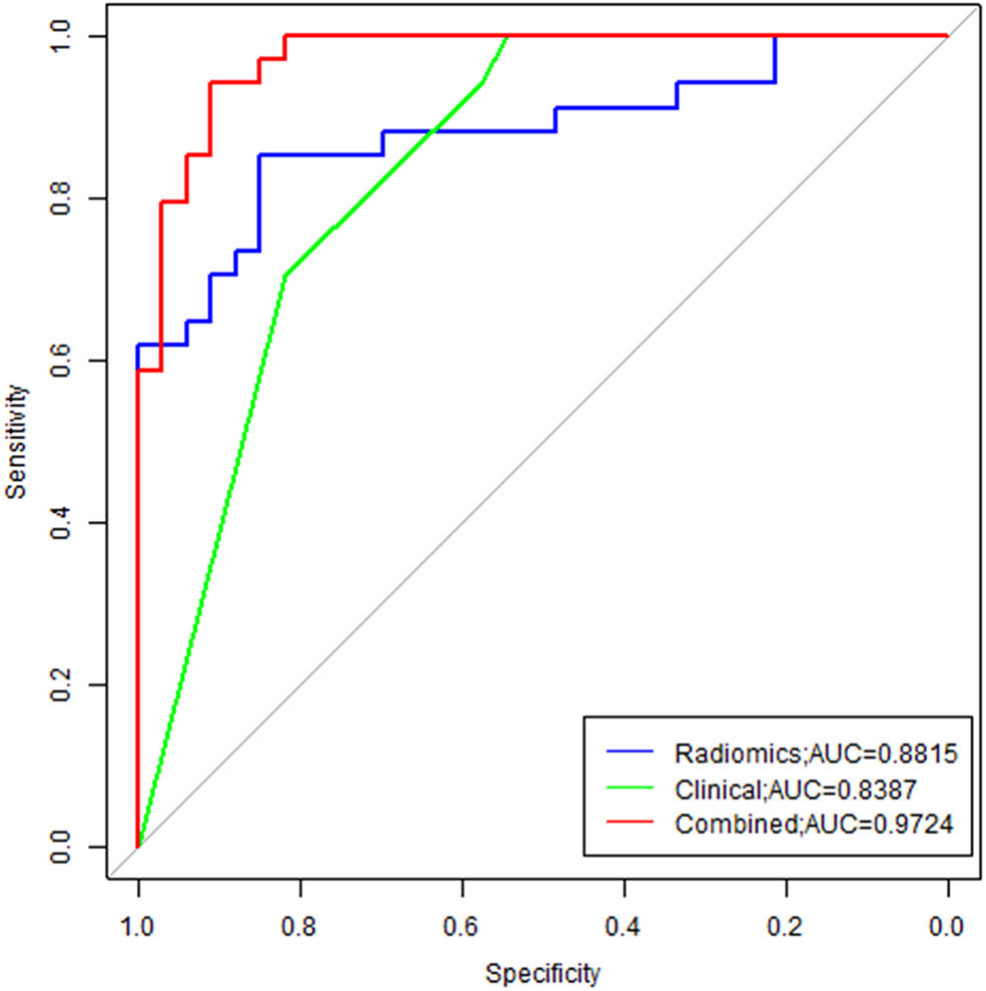
Comparison of ROC between three models. Radiomics model was marked with blue line, and the AUC was 0.8815; Clinical model was marked with green line, and the AUC was 0.8387; The combine model was marked with red line, the AUC was 0.9724.

In order to illustrate the potential ability for prediction of EGFR mutation, we compared the models developed by radiomics features, clinical variables, and combination of them. As we can see from Figure 6, the ROC curves showed the good performance and generalization for the model built by radiomics features, AUC for radiomics model was 0.8815. When the model built by the both radiomics features and clinical variables, the AUC was 9724, which was significantly higher than the single radiomics model and the single clinical variables model, this indicates that the combine model showed best performance to predict EGFR gene mutations.

### Analysis of an Individualized Prediction Model

**Figure 7:**
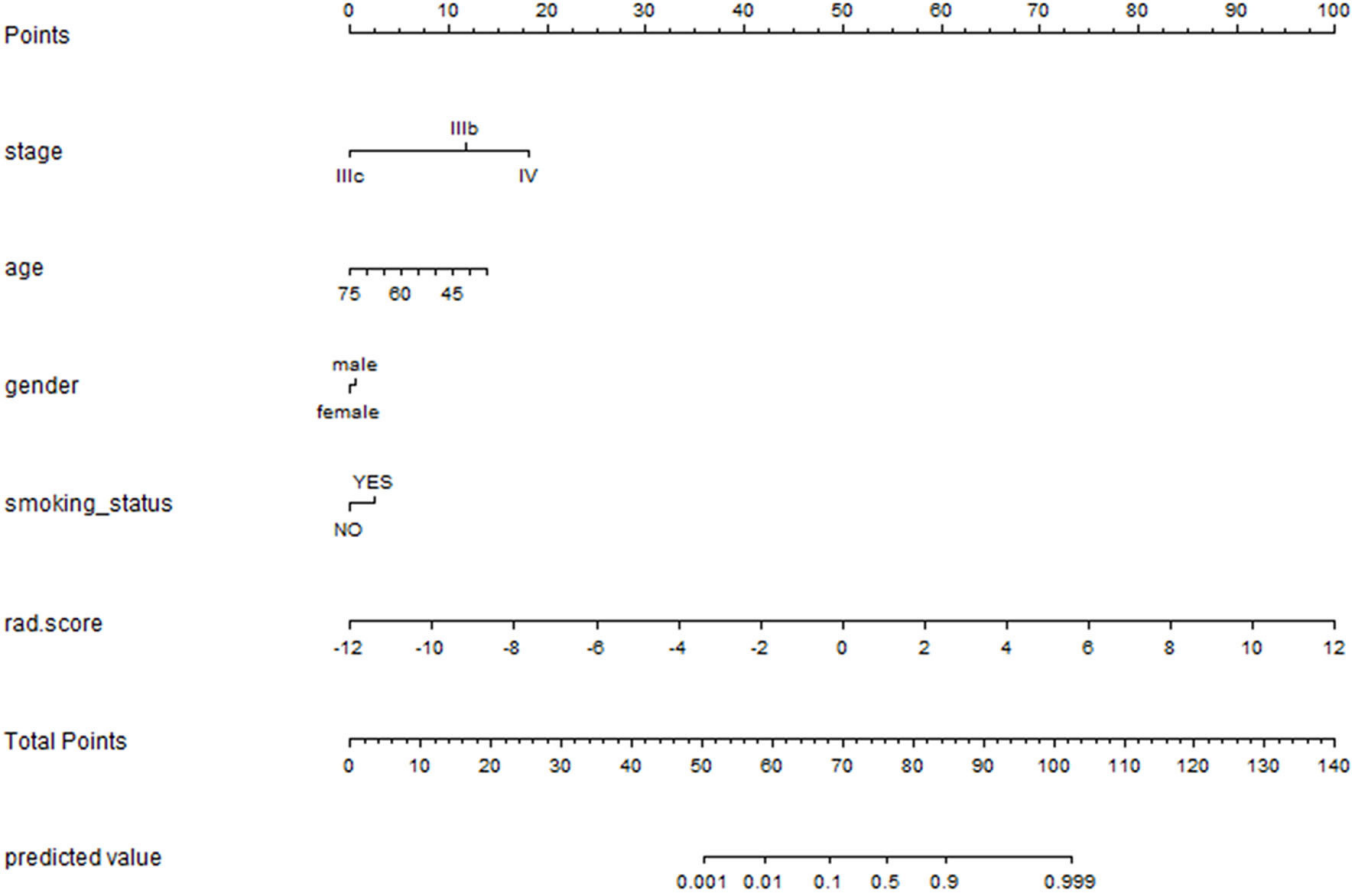
The nomogram was depicted to present the relationship between radiomic features and clinical features and visually show the potential ability individually. The nomogram was built with the stage, age, gender, smoking status and rad_signatures.

Multivariate logistic regression analysis identified the clinical stage, age, gender, smoking status, and Rad_signature as independent predictors. The individualized EGFR mutation prediction model that consisted of the above independent predictors was visualized by the nomogram (Figure 7).

## Discussion

The malignant degree of NSCLC is high and the 5-years survival rate of patients is low, exploring effective treatment has become a widespread concern in recent years. Because of the high expression of epidermal growth factor in 40–80% of NSCLC patients, the molecule can be used as a target molecule for specific targeting therapy when the patient detects an EGFR mutation. So, the purpose of this study was to analyze the imaging features and clinical characteristics of 67 patients, to explore the correlation between chest enhanced CT imaging features and EGFR mutation status, to construct a prediction model combined with imaging features and clinical characteristics, and to draw a clinical predictive nomogram.

Early studies have shown inconsistent findings regarding the correlation between CT findings and EGFR mutation status in patients with NSCLC (14–16). According to a study by Zhou et al. (17), EGFR mutants and wild type were no difference in CT morphological features of lung tumors. In contrast, Rizzo et al. (18) reported that EGFR mutations are closely related to air bronchography, pleural retraction, lesion size, and presence or absence of fibrosis. In recent years, with the deepening of research, a large number of studies by Ozkan et al. (19) have demonstrated the potential of CT-based quantitative imaging features in identifying EGFR mutants and wild type in NSCLC. Mei et al. (20) also found that CT texture features are not only related to EGFR mutation status, but also can further distinguish patient mutation sequences (18–20).

In our study, a total of 67 patients with NSCLC were included in the analysis of clinical features, imaging findings and EGFR mutations. We extracted 849 features for each patient, which can provide us with more details and help us complete the evaluation of the lesion more comprehensively and accurately, it's very important. In response to EGFR mutations, we constructed three predictive models (based on patient clinical, radiomics, combined radiomics and clinical), the AUC values were 0.8387, 0.8815, and 0.9724, respectively. The results of this study are consistent with the study of Liu et al. (8), indicating the imaging features are associated with EGFR mutation status in NSCLC patients. Different from Liu et al., 849 features were extracted for each patient in this study, while Liu's study only extracted 219 features. The increase in the number of features provides us with more valuable information and greatly improved the performance of the model (0.709 vs. 0.972). we also perform a consistency test on the extracted features, only highly stable features (ICC > 0.75) can be used for subsequent feature screening to ensure the stability of the study. In addition, we developed a radiomic nomogram based on a multivariable logistic analysis and radiomic signature (Rad_signature) to represent the possibility for each patient, these are not available in Liu et al. We also can find that in our study the image features used to construct the predictive model are texture, wavelet features, the results were not match Hsu et al. (21) previously reported that tumor size are correlation, which may be due to previous research based on only one level of tumor to extract tumor size characteristics (most the larger tumor areas), and our study focused on the size of the entire tumor extraction, leading to the emergence of differences. When compare the three models, we can find that the model combining imaging features and clinical has higher diagnostic efficiency than the single image model or clinical model; This also indicates that the image features and clinical features may reflect different valuable prediction information, the combination of two can complement the information and improve the prediction ability.

Compared with recent studies by Tu et al. (22) and Zhao et al. (23), the diagnostic efficacy of this study is significantly higher. This may be because our study is based on enhanced CT images. Compared with plain images, enhanced images can reflect tumors more valuable information on blood supply, internal lesions and more. Secondly, in some CT images of patients with lung cancer and atelectasis, enhanced images can more accurately delineate the edge of the lesion, reduce the impact on imaging features, and improve the diagnostic efficacy of the model for EGFR mutations in non-small cell lung cancer. In addition, Tu et al. and Zhao et al. Studies have lots of patients, but the sample size in our study is small, and the samples from the same institution will also have an impact on the results.

Although this study has achieved high diagnostic performance, the study still has some limitations. First, according to the Transparent Reporting of a multivariable prediction model for Individual Prognosis Or Diagnosis (TRIPOD) (24), in addition to internal cross-verification, the developed prediction model needs to evaluate the performance in external data to avoid overfitting. In this study, due to the small sample size, we have only performed internal cross-validation, and the independent model assessment could not be performed, which may have an impact on the true diagnostic performance of the prediction model. The next study requires large sample data from multi-center and independent model validation to confirm our findings. Secondly, the 5-mm slice thickness image is used in this study, due to the acquisition parameter have influence in the feature stability (25, 26), it can also lead to an inaccurate evaluation. In the future research, a prospective study is adopted, and the comparison between the models can be more facilitated by the standardized image acquisition and reconstruction algorithm, so as to improve the generalization of the model and the clinical application capability.

Our preliminary study indicates CT radiomics features are associated with EGFR mutation in patients with NSCLC, and CT imaging features of lesions on pretreatment may function as non-invasive biomarkers for improve stratification in patients with EGFR mutation and EGFR wild.

## Data Availability Statement

All datasets generated for this study are included in the article/supplementary material.

## Ethics Statement

Ethical review and approval was not required for the study on human participants in accordance with the local legislation and institutional requirements. Written informed consent for participation was not required for this study in accordance with the national legislation and the institutional requirements.

## Author Contributions

BG and SW: project design. BG: project support. SW and GS: data collection. SW, GS, and JM: data analysis. SW: manuscript writing. All authors: final approval of manuscript.

## Conflict of Interest

The authors declare that the research was conducted in the absence of any commercial or financial relationships that could be construed as a potential conflict of interest.
